# Metal Nanoclusters with Synergistically Engineered Optical and Buffering Activity of Intracellular Reactive Oxygen Species by Compositional and Supramolecular Design

**DOI:** 10.1038/s41598-017-05156-9

**Published:** 2017-07-20

**Authors:** B. Santiago-Gonzalez, A. Monguzzi, M. Caputo, C. Villa, M. Prato, C. Santambrogio, Y. Torrente, F. Meinardi, S. Brovelli

**Affiliations:** 1 Dipartimento di Scienza dei Materiali, Università degli Studi Milano-Bicocca, via R. Cozzi 55, 20125 Milano, Italy; 2 Dipartimento di Patofisiologia e dei Trapianti, Università degli Studi di Milano, Fondazione IRCCS Cà Granda Ospedale Maggiore Policlinico, Centro Dino Ferrari, Via Francesco Sforza 35, 20122 Milano, Italy; 3 Istituto Italiano di Tecnologia, Via Morego 30, 16163 Genova, Italy; 4 Dipartimento di Biotecnologie e Bioscienze, Università degli Studi Milano-Bicocca Piazza della Scienza, 2 20126 Milano, Italy

## Abstract

Metal nanoclusters featuring tunable luminescence and high biocompatibility are receiving attention as fluorescent markers for cellular imaging. The recently discovered ability of gold clusters to scavenge cytotoxic reactive oxygen species (ROS) from the intracellular environment extends their applicability to biomedical theranostics and provides a novel platform for realizing multifunctional luminescent probes with engineered anti-cytotoxic activity for applications in bio-diagnostics and conceivably cellular therapy. This goal could be achieved by using clusters of strongly reactive metals such as silver, provided that strategies are found to enhance their luminescence while simultaneously enabling direct interaction between the metal atoms and the chemical surroundings. In this work, we demonstrate a synergic approach for realizing multifunctional metal clusters combining enhanced luminescence with strong and lasting ROS scavenging activity, based on the fabrication and *in situ* protection of Ag nanoclusters with a supramolecular mantle of thiolated-Au atoms (Ag/Au-t). Confocal imaging and viability measurements highlight the biocompatibility of Ag/Au-t and their suitability as fluorescent bio-markers. ROS concentration tests reveal the remarkable scavenging activity of Ag-based clusters. Proliferation tests of cells in artificially stressed culture conditions point out their prolonged anti-cytotoxic effect with respect to gold systems, ensuring positive cell proliferation rates even for long incubation time.

## Introduction

Metal nanoclusters, owing to their size- and shape-tunable electronic properties^1^, ultra-large surface-to-volume ratios, low toxicity^2^ and to the flexibility of their physical properties *via* surface functionalization^3–7^, are receiving growing attention in several technological areas, spanning from solid state lighting^8^, solar cells^9^ and sensors^10, 11^ to photo-catalysis^12, 13^ and biomedical applications^10, 14–20^. The archetype metal nanoclusters are gold-based systems, whose luminescence properties can be controlled through a variety of approaches including, quantum confinement effects^1^, ligand-to-metal electron transfer^3, 21, 22^, controlled surface complexation^4, 23^, ligand-controlled formation of super-cluster architectures^24^ and through so-called aggregation induced emission between thiolate-protected clusters^25^. In addition to this, Au clusters have recently been demonstrated to scavenge intracellular reactive oxygen species (ROS), which are highly reactive compounds that are typically formed as a by-product of the cellular oxygen metabolism and play important roles in cell signalling and homeostasis^26^. However, when overexpressed in stressed conditions (i.e. chemical intoxication, UV exposure, overheating), ROS lead to accelerated cell ageing and, in extreme cases, to premature cellular death^27^. Although the exact mechanism of ROS scavenging by Au clusters is not fully understood and it is likely associated with the ability of the thiol capping ligands to buffer oxygen radicals^28–30^, this newfound ability of metal clusters to reduce oxidative stress is particularly beneficial for biomedical theranostics as it opens the way to novel multifunctional optical probes synergistically engineered for diagnostic and, potentially, therapeutic applications.

One possible strategy for achieving higher ROS harvesting performances would be to use clusters of more reactive metals than gold, such as silver. Ag based nanomaterials are known for their antibacterial properties, being their toxicity commonly associated to the release of Ag^+^, which is at the basis of the strategies for developing new antibiotics^31–34^. However, the toxicity mechanism of Ag based nanoparticles may not be directly applicable to all biological systems, i.e. in mammaliam cells probably due to the more complex sub-cellular organization. On the other hand, a comparative study of the biologic activity of Ag clusters with reduced (Ag^0^) or oxidized (Ag^+^) metallic core has shown stronger cytotoxicity of the first due to favoured release of Ag^+^ species leading to the expression of intracellular ROS^35^.

One possible limitation to the use of Ag clusters as multifunctional luminescence markers is, however, that the emission efficiency of systems protected with small capping molecules, such as thiols, is typically very low^36–38^. In order to address this issue, Ag clusters capped with bulky biomolecules^39–41^ and polymers^42^, which protect the metal cores from luminescence quenchers^43^, have been realized. The steric encapsulation of the metal cores, however, increases the hydrodynamic size of the clusters, which could limit their permeability in subcellular imaging^44^ and, more detrimental for ROS scavenging, prevents direct contact between the cluster and its chemical surroundings.

In order to simultaneously achieve enhanced optical and anti-cytotoxic performances, it is therefore paramount to develop synergistic passivation strategies that protect the excited states of the clusters while ensuring accessibility of ROS-sensitive surface metal core and sulfur functionalitity of the respecive thiol ligands to the intracellular environment. One possible approach could be supramolecular protection of the Ag clusters with a thin mantle of aggregated Au-thiolate complexes, as recently theoretically predicted for Au clusters by H. Hakkinen^45^ and validated experimentally by Xie and coworkers^4, 46^, who enhanced the luminescence efficiency of gold clusters by surface condensation of aggregated Au-thiolate oligomers. A core-shell approach, where a shell of fluorescent Ag_2_ and Ag_3_ clusters was added to Au clusters, has also been recently used for obtaining highly fluorescent core–shell particles^47^.

Taking inspiration from these pioneering studies, in this work, we develop a one-pot aqueous route for the synthesis and *in situ* protection of Ag clusters with Au-thiolate complexes, resulting in enhanced and spectrally pure blue emission and strong ROS scavenging activity. The synthetic rationale exploits the higher reactivity of silver with respect to gold^37^ that, when co-added with a reducing agent, drives the fast nucleation of Ag clusters that are successively decorated with Au-thiolate complexes. The progressive shelling of the silver clusters is confirmed by side-by-side structural and optical measurements on bare and Au-complexed Ag clusters (Ag/Au-t) during the synthesis reaction. Confocal imaging and viability measurements on NIH/3T3 fibroblast cells demonstrate the excellent biocompatibility of the Ag/Au-t clusters and their suitability as blue fluorescent markers. ROS concentration tests reveal, for the first time, the strong scavenging activity of both Ag and Ag/Au-t systems and confirm the ROS harvesting property of Au clusters^24^. Remarkably, proliferation tests of cells with artificially accelerated metabolic activity highlight the prolonged ROS buffering effect of Ag and Ag/Au-t clusters with respect to gold systems, ensuring positive and high cell proliferation rate even after 96 hours of incubation in stressed condition.

## Results

### Synthesis and Structure of complexed Ag clusters

**Figure 1:**
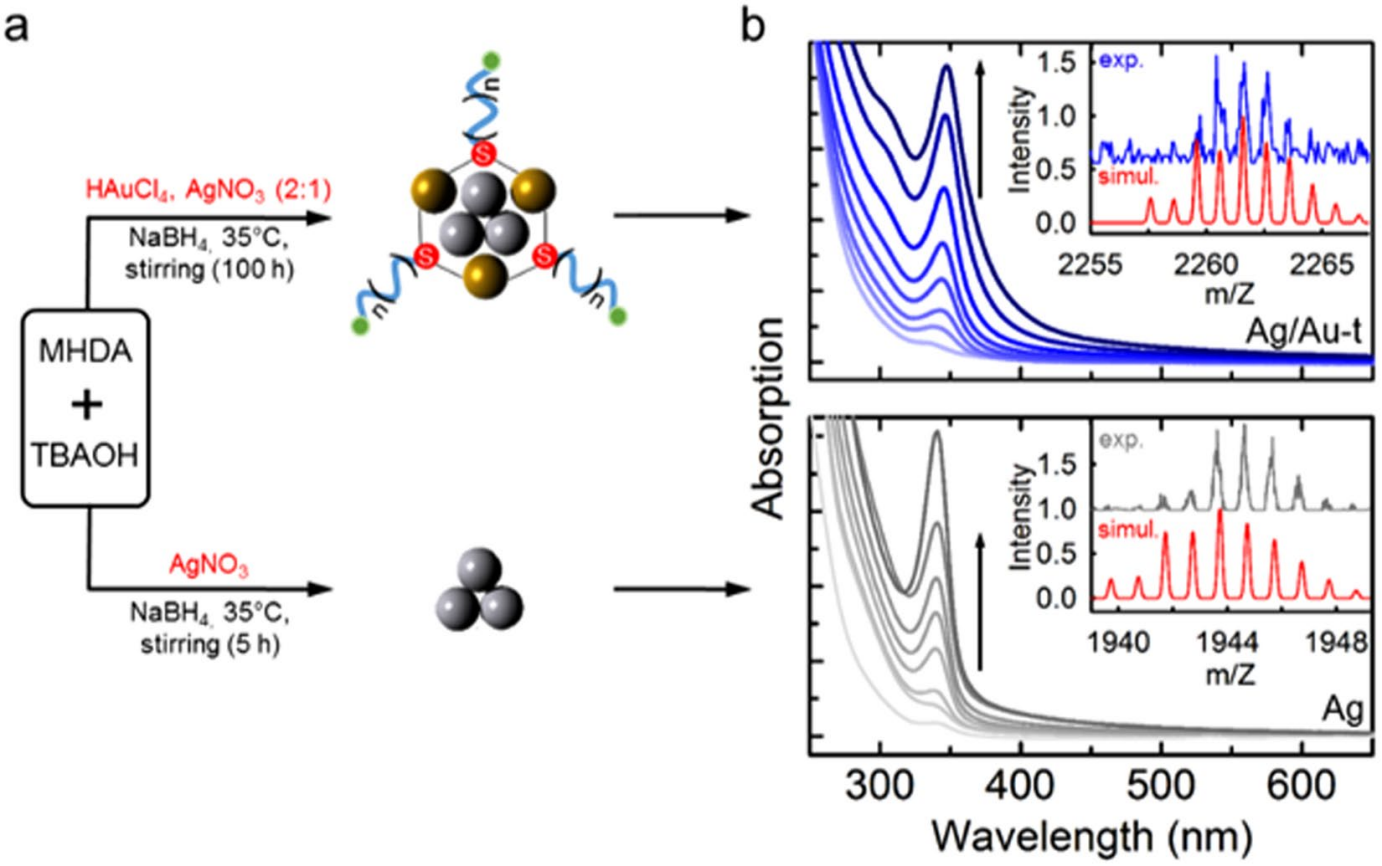
(**a**) Synthesis routes for the preparation of Ag clusters protected by Au-thiolate complexes (Ag/Au-t, top panel) and bare Ag clusters (bottom panel). In the schematic depictions of the two systems, the green circles correspond to carboxylic end-groups and the subscript n = 7. The molecular surfactants (tetrabutylammonium, TBA and 16-mercaptohexadecanoic acid, MHDA) are omitted for clarity. (**b**) Absorption spectrum of Ag/Au-t and Ag clusters aliquots of the reaction medium as function of time. The insets show the experimental and simulated isotopic mass distribution patterns of [Ag_3_Au_3_(MHDA)_3_(TBA)_2_H_2_]^+^ and [Ag_3_(MHDA)_2_(TBA)_4_(NO_3_)O]^+^ chemical species for the protected and bare clusters, respectively.

**Figure 2:**
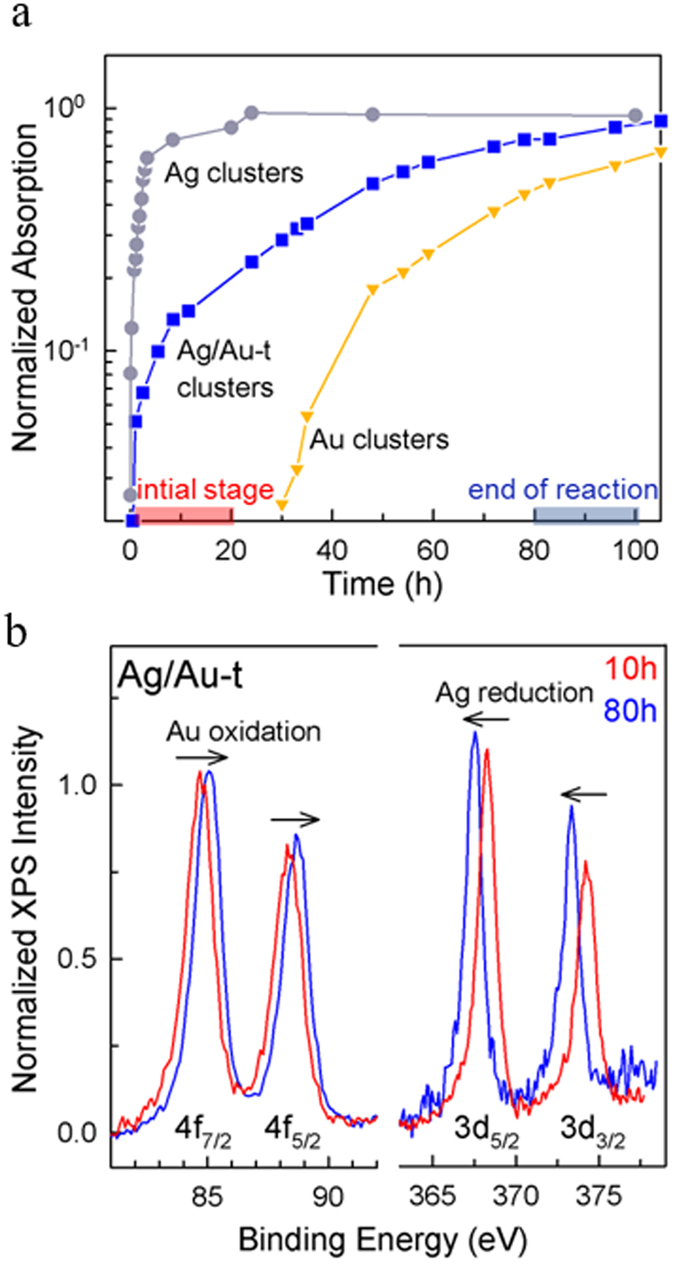
(**a**) Normalized absorption peak intensity measured on aliquots of the reaction medium for Ag (circles), Ag/Au-t (squares) and Au (triangles) clusters as a function of the reaction time. (**b**) XPS spectra of Au and Ag atomic transitions measured on Ag/Au-t clusters after 10 hours and 80 hours of the reaction time. The shift of the binding energies over time indicates the reduction of Ag atoms and the concomitant oxidation of Au atoms.

The Ag/Au-t capped with 16-mercaptohexadecanoic acid (MHDA)-tetrabutylammonium (TBA) salt were prepared according to the bottom-up route shown in Fig. 1a, which is based on the chemical reduction of the Ag precursor (AgNO_3_) in the presence of HAuCl_4_ (nominal Ag:Au ratio 1:2), mercapto-palmitic acids and tetrabutyl ammonium salts^48–50^. As control materials, we fabricated monometallic clusters by adopting the same reaction conditions as the synthesis of the Ag/Au-t systems, but introducing exclusively Ag or Au precursors in the reaction medium. The molar ratio between the capping ligands and metal precursors, which is a key parameter for tuning the cluster size^1^, has been kept constant for all syntheses. In order to monitor the nucleation of the clusters, we measured the optical absorption of aliquots extracted from the reaction medium over time, as shown in Fig. 1b. The time evolution of the absorption intensity for each system is reported in Fig. 2a. The Ag/Au-t clusters show a narrow absorption spectrum peaked at ~345 nm, whose intensity increases with the reaction time as the cluster population progressively grows. The spectral position of the absorption maximum remains unchanged at all stages of the synthesis, which indicates the formation of clusters with identical dimensions according to the typical size-focused growth of metal clusters^51–53^. The absorption spectrum of the Ag/Au-t systems is essentially identical to the Ag clusters, which suggests that both routes lead to the nucleation of Ag cores of comparable size corresponding to ca. three silver atoms^54^. This assignment is confirmed by electrospray-ionization mass spectrometry (ESI-MS) analysis (Fig. S2), which indicates that bare silver clusters are composed of three Ag atoms capped by two MHDA ligands. The experimental and simulated isotopic distribution pattern corresponding to the [Ag_3_(MHDA)_2_(TBA)_4_(NO_3_)O]^+^ species is shown in the inset of Fig. 1b (lower panel). On the other hand, the ESI-MS spectrum of the Ag/Au-t systems shows two peaks at 2245 *m*/*z* and 2261 *m*/*z*, respectively assigned to the [Ag_3_Au_3_(MHDA)_2_(TBA)_3_(BH_4_)_2_]^+^ and the [Ag_3_Au_3_(MHDA)_3_(TBA)_2_H_2_]^+^ species. The inset of Fig. 1b (top panel) reports the experimental spectrum of the latter structure together with the corresponding simulated pattern. Notably, no feature observed for the bare Ag sample is found in the spectrum of the complexed clusters (Fig. S2), which indicates that the Ag/Au-t ensemble is composed by nearly monodispersed Ag_3_ cores decorated with Au-thiolate units.

The absorption spectrum of the control Au clusters is markedly different from both Ag and Ag/Au systems, with two broader peaks at 390 nm and 422 nm (Fig. S1). The absence of such absorption features in the spectrum of the Ag/Au clusters indicates that the population of Au clusters in the bimetallic sample is negligible. The comparison between the absorption spectra of the three systems suggests that, in the bimetallic reaction, the nucleation of the Ag cores is favoured over the synthesis of the Au clusters.

This picture is supported by the comparison of the formation kinetics of the three cluster types shown in Fig. 2a, which we monitored through the time evolution of the respective absorption amplitude. The monometallic Ag clusters show a rapid growth of their absorption spectrum at the very early stage of the synthesis, which reaches saturation in approximately three hours, indicating the conclusion of the nucleation reaction. Conversely, the formation of monometallic Au clusters is much slower, with the characteristic absorption features being detectable only after over 24 hours of reaction. This striking difference between the growth kinetics is ascribed to the lower reduction potential of silver (Ag^+^/Ag, ~0.8 V) with respect to gold (Au^+3^/Au, ~1.0 V), which accelerates the formation kinetics of the Ag clusters with respect to the Au ones^37^. Notably, the synthesis of the Ag/Au systems follows a fast trend similar to the monometallic Ag clusters, suggesting that the bimetallic reaction is initially driven by the nucleation of the Ag cores followed by the preferential incorporation of Au atoms with respect to homonucleation of Au clusters.

In order to gather deeper insights into the mechanism of the bimetallic reaction, we performed X-ray photoelectron spectroscopy (XPS) measurements on two Ag/Au cluster aliquots, taken respectively after 10 hours and after 80 hours of reaction time. In both cases, the samples were thoroughly washed by precipitation prior to the XPS analysis. Figure 2b reports the XPS spectrum of Ag/Au-t clusters showing the binding energies of Au 4 f and Ag 3d electrons. At the initial stage of the reaction, the binding energies obtained for silver are E_Ag_(3d_5/2_) = 368.2 eV and E_Ag_(3d_3/2_) = 374.2 eV, consistent with the values found for small-sized thiolated-Ag clusters^55^, bimetallic Ag-Au systems^56^, as well as with the optical absorption data in Fig. 1b. For gold, the initial binding energies are E_Au_(4f_7/2_) = 84.7 eV and E_Au_(4f_5/2_) = 88.5 eV (Fig. 2b), in agreement with the energies of thiolated Au atoms^57^. After 80 hours, the XPS spectrum shows a shift of the binding energies of both metals: The silver energies decrease to E_Ag_(3d_5/2_) = 367.5 eV and E_Ag_(3d_3/2_) = 373.3 eV, indicating the reduction of the Ag atoms upon removal of thiol ligands from the cluster surface. The Au binding energies increase to E_Au_(4f_7/2_) = 85.1 eV and E_Au_(4f_5/2_) = 88.8 eV, indicating the oxidation of metallic gold to Au^+1^ atoms, which is consistent with their final location on the Ag cluster surfaces and with the formation of oligomeric Au-thiolate complexes^57^. This is supported by the binding energies observed in the reference monometallic Ag systems [E_Ag_(3d_5/2_) = 367.7 eV and E_Ag_(3d_3/2_) = 373.7 eV, Fig. S3], which are very close to those of the Ag/Au-t case, although slightly shifted to higher values due to the presence of the thiol capping on the surface^55^. The binding energies measured for the monometallic Au clusters are significantly lower [E_Au_(4f_7/2_) = 84.3 eV and E_Au_(4f_5/2_) = 88.5 eV, Fig. S3]^57^, thus confirming the absence of isolated gold clusters at the end of Ag/Au-t clusters synthesis. The gradual complexation of the Ag cores by thiolated-Au species is further confirmed by the quantitative analysis of the XPS data. Specifically, the Ag:Au atomic ratio in the aliquot extracted after 10-hours reaction is 1:0.5, with a concentration of gold atoms four times lower than the nominal feeding ratio of 1:2. This indicates that, at the early stages of the synthesis, most of the gold atoms are not attached to the Ag clusters and are thus washed away during purification. On the other hand, the Ag:Au atomic ratio after 80 hours of reaction is 1:1.5, which is close to the nominal concentration of precursors and indicates that gold atoms have been incorporated in the cluster architecture and thus unaffected by the purification procedure.

### Photoluminescence Properties

**Figure 3:**
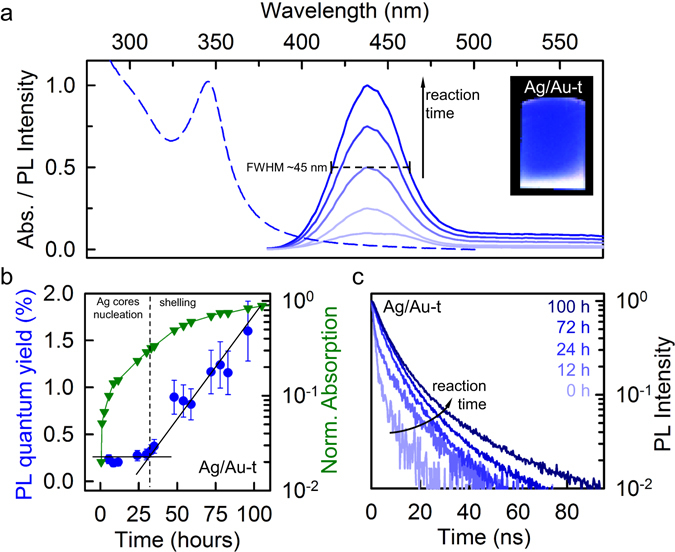
(**a**) Absorption and PL spectrum of protected Ag clusters (Ag/Au-t) dispersion in water. The intensity of the emission increases with the reaction time. The inset is a digital picture of the dispersion under UV lamp excitation. (**b**) PL quantum yield (circles), normalized absorption (triangles) and (**c**) time-resolved PL decay traces at 435 nm as function of the reaction time. All measurements were performed at room temperature using a 355 nm pulsed laser as excitation source, with an excitation fluence of 130 μJ/cm^2^.

The progressive condensation of thiolated Au species on the Ag cluster surfaces has beneficial effects on their emission efficiency, as highlighted by side-by-side continuous-wave and time-resolved photoluminescence (PL) measurements performed during the synthesis reaction. Figure 3a shows the evolution of the PL spectrum of Ag/Au-t clusters excited at 355 nm (130 μJ/cm^2^) as a function of the reaction time, together with the absorption spectrum after 80-hours reaction. The PL spectrum consists of a narrow peak at 435 nm that grows over time without any appreciable spectral modification, with full width at half maximum of ~45 nm at all reaction stages (Fig. 3a).

Importantly, the increase of the PL intensity during the synthesis is not only due to the growing number of clusters in the sample, but also to the progressive enhancement of their PL quantum yield. This effect is shown in Fig. 3b, where we report the evolution of the PL efficiency and sample absorbance over time, which further enables us to monitor the progress of the Au-thiolate passivation process. Specifically, in the first 30 hours of reaction, which correspond to the time required for nearly concluding the synthesis of the bare Ag cores (Fig. 3b triangles and Fig. 2a), the PL efficiency is nearly constant and relatively low, in agreement with previous reports on Ag clusters protected with small thiol ligands^36–38^. In this time interval, the growth of the PL signal is therefore due to the increasingly larger number of clusters in solution, consistent with over 10-fold increase of the ensemble’s absorbance. After ca. 30-hours reaction, when the absorption trend of Ag/Au-t clusters is markedly slower (3-fold increase until reaction end) and that of the monometallic Ag clusters is essentially constant (Fig. 2a), the PL quantum yield undergoes progressive increase, resulting in ~10-fold enhancement in the final products with PL efficiency ~2% (Fig. 3b). This effect is consistent with the gradual protection of a steady population of Ag clusters by Au-thiolate complexes that passivate non-radiative quenching channels. Accordingly, the PL decay dynamics of Ag/Au-t clusters becomes progressively slower, as shown in Fig. 3c. We notice that other mechanisms could, in principle, lead to enhanced PL, such as the intercalation of Au atoms between the early-formed Ag cores and their capping thiols, or the substitution of Ag atoms with thiolated Au species. Both these mechanisms would, however, result in significant alteration of the electronic structure of the clusters, either due to increase of their size by incorporation of additional atoms^10^, or to the modification of their chemical composition^38^, in disagreement with the optical absorption and PL spectra in Figs 1b and 3a. We note that the final PL quantum yield of our Ag/Au-t clusters is ~2%, which indicates that the shelling is incomplete. This is, however, beneficial for the applicability of Ag/Au-t clusters as multifunctional luminescent probes in cellular theranostics, as it increases the PL efficiency enough for fluorescence imaging, while still allowing direct interaction between the metal cores and the intracellular environment. This enables, as we demonstrate below, to exploit the strong ROS-scavenging activity of the thiolated silver clusters.

### Cellular Imaging and ROS essays

**Figure 4:**
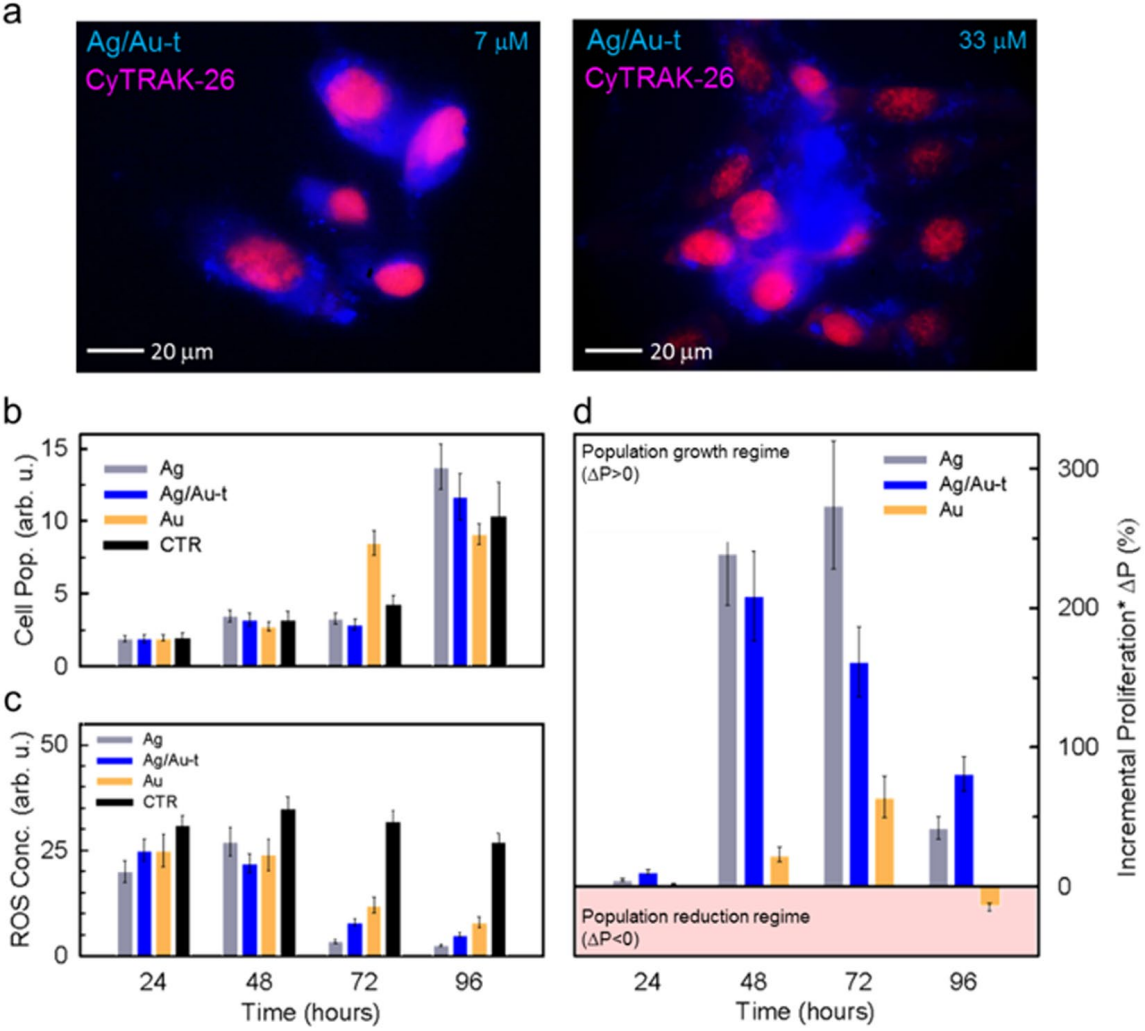
(**a**) Confocal fluorescence microscope images of fixed NIH/3T3 fibroblast cells co-stained with Ag/Au-t clusters (blue) and CyTRAK-26 dye (red) under UV excitation at increasing cluster concentration (7 µM and 33 µM). (**b**) Cytotoxicity test and (**c**) ROS concentration level test on NIH/3T3 cells stained with 7 µM of Ag, Ag/Au-t and Au clusters, taken at four time-points during cells proliferation (24 h, 48 h, 72 h and 96 h). (**d**) Cellular proliferation test on cells in artificially stressed culture conditions by adding the metabolic accelerator menadione. The histogram shows the incremental cell proliferation of cells stained with the metal clusters calculated with respect to the unstained control culture.

To experimentally validate the applicability of our proof-of-concept Ag/Au-t clusters as fluorescent bio-markers, we performed *in vitro* imaging experiments on NIH/3T3 fibroblast cells. Figure 4a reports representative RGB (red-green-blue) images of NIH/3T3 cells co-stained with the red emitting dye CyTrak-26 - for selectively marking the nucleus - and with increasing concentration of Ag/Au-t clusters that disperse in the cytoplasm as they are not functionalized with target-specific ligands. We notice that the Ag/Au-t clusters do not aggregate unless at high concentration (33 μM, obtained with a 1:10 dilution of the mother solution, Fig. 4a right panel).

After having confirmed the effectiveness of the supramolecular shelling in rendering Ag clusters suitable as fluorescent bio-probes, we proceeded with the investigation of the effects of Ag/Au-t on the cellular biology in direct comparison with their unshelled counterparts. With this aim, we performed the 3-(4,5-dimethylthiazol-2-yl)-2,5-diphenyltetrazolium bromide (MTT) essay and monitored the evolution of ROS over time. The results of the MTT test in standard culture conditions reported in Fig. 4b indicate that neither Ag nor Ag/Au-t clusters affect the cell viability. The viability tests reveal cell proliferation rates comparable for stained cells with respect to untreated cells for the whole duration of the experiment (96 hours), outlining the high biocompatibility of these material systems. As previously demonstrated^24^, the culture viability is unaffected also by Au clusters, which however, slow down the cell proliferation at long incubation times. The improved cell viability suggests that Ag and Ag/Au-t clusters can also exert a protective role even in standard cell culture conditions when ROS-dependent mechanisms of cell growth regulation occur, as showed by the ROS concentration tests reported in Fig. 4c. The ROS level in a standard cell culture is associated with the acute stress induced by the initial cell detachment from the culture vessel required to perform the test (24 hours to 48 hours)^58^ and to the negative regulation of the cell culture proliferation - also known as “contact inhibition” - when grown to confluence (72 hours to 96 hours)^59^. Therefore, this continuous stress condition results in a constant ROS concentration level for the unstained control culture at each time point considered. Conversely, the presence of metal clusters is able to counterbalance the ROS overproduction in stained cultures. In particular, all clusters show comparable ROS-scavenging abilities in the first 48 hours, reducing the level of oxidants agents produced upon cell detachment to similar values. At longer times, when the occurrence of cell confluence induces additional stress, only Ag and Ag/Au-t systems still operate effectively, turning out in a rapid cell growth even at 96 hours from seeding (Fig. 4b). The Au clusters, despite being able to decrease the overall ROS concentration, show a weaker ability to compensate this additional stress with respect to their Ag and Ag/Au-t counterparts, thus causing a slower proliferation (Fig. 4b). Beside confirming recent results on Au clusters^24^, these findings demonstrate, for the first time, the ROS scavenging activity of thiolated Ag clusters. These results are further confirmed by independent experiments using the ROS-Glo™ H_2_O_2_ Assay (Supplementary Fig. S4), which demonstrate that both Ag and Ag/Au-t systems strongly reduce the concentration of H_2_O_2_ also in aqueous solution. Importantly, the scavenging activity of Ag clusters is found to be stronger than for Au systems in agreement with the higher reactivity of silver atoms. Specifically, in Au-clusters stained cells, the ROS concentration drops by ~70% in 96 hours, whereas in cells marked with Ag-based clusters over 90% reduction is achieved already after 72 hours. According to the partial passivation effect of Au-thiolate of the Ag cores, which accounts for the enhanced PL efficiency with respect to bare Ag clusters shown in Fig. 3, the ROS scavenging activity of the shelled systems is slightly lower than for the bare Ag clusters, yet still more effective than for the Au clusters. Notably, PL measurements on Ag/Au-t clusters in water as a function of time (Fig. S5) reveal that their emission efficiency is essentially unaffected by ROS concentrations as high as 0.5 mM for over five hours of exposure and undergoes only 40% drop for very high ROS content (5 mM), which suggests that these emitters could be employed for bio-imaging measurements also in harsh conditions.

It is worth pointing out that these encouraging findings cannot be considered as a proof of the cluster scavenging abilities in severe oxidative stress conditions that trigger cell death^60^, since the low levels of intracellular ROS recorded are only symptomatic of signal transduction pathways involving the responses to growth factors, hypoxia, and other receptor-ligand systems^61^. Therefore, in order to verify that this newfound ROS buffering effect of Ag and Ag/Au-t clusters has significant implications, we studied the cellular proliferation in highly stressed conditions upon addition of the metabolic accelerator menadione, which induces intense toxic oxidative stress mimicking the condition of *in vivo* tissue injury and mitochondrial DNA damage^62^. Figure 4d reports the incremental proliferation ΔP = 100 × (P_stain_ − P_CTR_)/P_CTR_ of cluster-stained NIH/3T3 fibroblasts (P_stain_) in the presence of menadione. ΔP was calculated with respect to the population of the unstained control culture (P_CTR_) incubated in identical conditions. In agreement with the stronger ROS scavenging effect of Ag and Ag/Au-t clusters with respect to Au clusters (Fig. 4c), Fig. 4d shows markedly increased proliferation of the respective cell cultures, with ΔP > 200% within the first 48 hours vs. ΔP ~ 25% for cells containing Au clusters. Furthermore, Ag-based clusters show longer lasting ROS buffering effect than Au clusters, resulting in significant cell growth (ΔP > 50%) even after 96-hours incubation, when cells stained with Au clusters show a mild proliferation drop.

## Conclusions

In summary, we have demonstrated a synergic strategy for realizing multifunctional metal clusters combining amplified and prolonged anti-cytotoxic activity and spectrally pure photoluminescence for applications in bio-diagnostics and conceivably cellular therapy. The approach consists in the use of Ag clusters with stronger reactivity than Au with oxygen radicals and their partial passivation with a mantle of Au-thiolate complexes that reduce non-radiative luminescence quenching channels, while still preserving sufficient accessibility to enable direct interaction of the thioated Ag core with its chemical environment. As a result, these complexed clusters can be used in cellular theranostics as fluorescence markers and intracellular scavengers of cytotoxic species with beneficial effects on the cellular viability. We note that the reported proof-of-principle Ag/Au-t clusters are not optimized in terms of the gold coating thickness or the choice of the capping ligand, and further improvements in the photoluminescence quantum efficiency and ROS scavenging activity might therefore be expected by adjusting the silver core coverage and functionalization. The strategy demonstrated here for Au-passivated Ag clusters is not metal or size specific and might, in principle, be extended to other compositions, so as to achieve biocompatible multifunctional metal nanoclusters with diagnostic and potentially therapeutic ability.

## Materials and Methods

### Materials

Typically, 3 mL of 16-mercaptohexadecanoic acid (Sigma Aldrich, 90%, 0.0347 M) (with the necessary volume of tetrabutyl ammonium hydroxide solution, technical, ~40% in H_2_O,until neutralization) were added to a 5.4 mL of ultrapure water under vigorous stirring. Next, 500 µL of HAuCl_4_·3H_2_O(Sigma Aldrich, 99.999% trace metal bases) solution (0.0147 M) were added to the mixture followed by 250 µL of AgNO_3_(Sigma Aldrich, 99.9999% trace metal bases) solution (0.0147 M) and stirred for five minutes, after which 750 µL of NaBH_4_(Sigma Aldrich, granular, 99,99% trace metal bases) 0.05 M were injected and immediately incubated at 35 °C. In a like manner, silver and gold monometallic clusters were prepared by adding 750 µL of the correspondent metal precursor solution (AgNO_3_ and HAuCl_4_·3H_2_O respectively) in order to maintain the ligand-to-metal ratio constant. The mixtures were left to react until no changes in absorption spectra were observed. Then, the samples were purified by precipitating them with isopropanol 3 times followed by their resuspension in ultrapure water.

### Optical Measurements

Absorption spectra were collected with a Cary Varian 50 spectrophotometer at normal incidence in 1 mm quartz Suprasil cuvettes (bandpass 1 nm). PL measurements were performed at room temperature using a Varian Eclipse spectrometer with a Xenon lamp as a continuous wave light source. To measure the PL quantum yield (QY), a solution of 9,10 diphenyl-anthracene in tetrahydrofuran (10^−6^ M) was used as fluorescence standard^63^. Time-resolved PL profiles were recorded with an Edinburg Instruments FLS 980 spectrometer using a 3.65 eV EP-LED as excitation source (pulse width 900 ps). All spectra were corrected for the instrumental response. Fluorescence micrographs were collected using a Canon EOS 400D camera coupled to a Nikon Ti-U inverted microscope. Samples were excited with a Xenon lamp whose emission was spectrally filtered with a DAPI excitation filter (320–400 nm).

### Mass Spectrometry

Electrospray-ionization mass spectrometry (ESI-MS) experiments in positive-ion mode were performed on a hybrid quadrupole/time-of-flight (qTOF) instrument equipped with a nanoelectrospray ion source (AB Sciex, ForsterCity, CA, USA). The samples were centrifuged at 14.000 × g for 5 minutes in order to remove insoluble materials. The resulting supernatants were diluted 1:1 in acetonitrile and infused by borosilicate-coated capillaries of 1 μm internal diameter (Thermo Fisher Scientific, Waltham, MA USA). The main instrumental parameters were: ion-spray voltage 1.1 kV; curtain gas 20 PSI; declustering potential 80 V. The recorded spectra were averaged over 1 minute acquisition time. The simulation of the isotopic distribution was performed by the software IsoPro 3.1 (Software Tools for Mass Spectrometry).

### X-ray Photoelectron Spectroscopy

XPS measurements were performed using a Kratos Axis Ultra^DLD^ spectrometer with monochromatic Al Kα source operated at 15 kV and 10 mA. The specimen for XPS was prepared by drop casting 200 μl of a clean and concentrated solution onto a silicon wafer. All the analyses were performed over an area of 300 × 700 microns. High-resolution analyses were carried out with a pass energy of 10 eV. In order to minimize the damaging of the organic ligands, fast spectra were collected on several areas of the specimen (acquisition time no longer than 8 minutes on each area) and successively averaged. The Kratos charge neutralizer system was used during data acquisition. Spectra have been charge corrected to the main line of the carbon 1 s spectrum set to 284.8 eV (C-C bond). Spectra were analyzed using Casa XPS software (version 2.3.16). From a quantitative point of view, it is possible to estimate the Au:Ag atomic ratio in the specimen by calculating the ratio between the areas under the Au-4f and Ag-3d profiles, after normalization to the relative sensitivity factors (RSF), which depend on the cross sections for photoemission. For reference RSF (Au-4f) = 6.25 while RSF(Ag 3d) = 5.987. Such procedure yields a gold-to-silver ratio Au:Ag = 18:37 (~0.5:1) and 28:19 (~1.5:1) after 10 hours and 80 hours of reaction, respectively.

### Staining and Biocompatibility Tests

NIH/3T3 cells (ATCC® CRL-1658™) were thawed and plated on cell culture dish in DMEM high glucose (Gibco) supplemented with 10% FBS (Euroclone) for 48 hours before use. For immunofluorescence experiments, the cells were plated in a 12 multi-well plate at a density of 5 × 10^4^ cells/well; in each well a round cover glass slide was added in order to growth the cells on its surface and then visualise them by confocal microscopy. Before seeding, cells were stained with CyTRAK, following the producer protocol. Briefly, cells were centrifuged twice in serum free medium (400 g, 5 minutes) and then stained for 5 minutes with Dye Solution, as reported in datasheet. Cells were then rinsed twice in complete medium and seeded at the right density after checking fluorescence. After 24 hours the nanoparticles were added to the cells at the concentration indicated and, at 24, 48 and 72 hours, CyTRAK staining was performed in order to visualize cell nuclei and. Cover glass were then mounted with a 1:1 v/v glycerol-PBS solution.

### MTT Test

For proliferation experiments, cells were seeded in a 96 multiwell at a density of 3 × 10^3^ cells/well in triplicate; after 24 hours nanoparticles were added to the cell medium and at 24, 48 and 72 hours and 96 hours an MTT test was performed (Methylthiazolyldiphenyl-tetrazolium bromide, Sigma). Briefly: a 50 μg/ml MTT solution was added to the samples; after 4 hours of incubation at 37 °C the medium was removed, the converted dye solubilised with DMSO (dimethylsulfoxide, Sigma) and the absorbance measured at 560 nm (GloMax Discover, Promega). Unstained cells in growth medium were used as control condition. The proliferation test under oxidative stress has been performed by adding menadione (Sigma Aldrich, C_6_H_4_(CO)_2_C_2_H(CH_3_) to prompt cell apoptosis progressively increasing the intracellular level of ROS.

### ROS Test

For the evaluation of the Reactive Oxygen Species (ROS) produced and released *in vitro*, cells were seeded as described before for the MTT test; the analysis were performed 24, 48, 72 and 96 hours after the addition of clusters in culture medium; a ROS-Glo™ H_2_O_2_ Assay (Promega) was used, following the producer protocol. Unstained cells are reported as negative control. The Non-Lytic assay was applied and the relative luminescence units were measured by a plate reader (GloMax Discover, Promega). For solution studies, triplicate samples of metallic clusters in water (7 μM) were tested using ROS-Glo™ H_2_O_2_ Assay (Promega), following the manufacturers’ protocol. An opaque white 96 wells plate was loaded with 50 μl of each sample dispersed in H_2_O_2_ (5 mM) saturated water, followed by the incubation with H_2_O_2_ substrate solution and ROS-Glo detection solution. After 20 min incubation at room temperature, the luminescence was measure by GloMax Discover (Promega) plate reader. The luminescence was recorded continuously every 15 minutes for 5 hours.

## Electronic supplementary material

Supplementary materials
