# Supplementary material for: Metal Nanoclusters with Synergistically Engineered Optical and Buffering Activity of Intracellular Reactive Oxygen Species by Compositional and Supramolecular Design
Source: Sci Rep. 2017 Jul 20;7:5976. doi: 10.1038/s41598-017-05156-9 (PMC5519591; doi:10.1038/s41598-017-05156-9)
Supplement: Supplementary file 1 — Supplementary materials [file 41598_2017_5156_MOESM1_ESM.doc]

Supplementary Materials

**Metal Nanoclusters with Synergistically Engineered Optical and Buffering Activity of Intracellular Reactive Oxygen Species through Compositional and Supramolecular Design**

B. Santiago-Gonzalez*^1^, A. Monguzzi^1^, M. Caputo^1^, C. Villa^2^, M. Prato^3^, C. Santambrogio^4^, Y. Torrente ^2^, F. Meinardi ^1^, and S. Brovelli* ^1^

*^1^ Dipartimento di Scienza dei Materiali, Università degli Studi Milano-Bicocca, via R. Cozzi 55, 20125 Milano, Italy.*

*^2^ Dipartimento di Patofisiologia e dei Trapianti, Università degli Studi di Milano, Fondazione IRCCS Cà Granda Ospedale Maggiore Policlinico, Centro Dino Ferrari, Via Francesco Sforza 35, 20122 Milano, Italy.*

*^3^Istituto Italiano di Tecnologia. Via Morego 30, 16163 Genova, Italy.*

*^4^ Dipartimento di Biotecnologie e Bioscienze, Università degli Studi Milano-Bicocca Piazza della Scienza, 2 20126 Milano*

*Correspondence to be addressed to: [beatriz.santiago@unimib.it](mailto:beatriz.santiago@unimib.it), [sergio.brovelli@mater.unimib.it](mailto:sergio.brovelli@mater.unimib.it)

1. **Synthesis of Au clusters**

The absorption spectrum of the control Au clusters showed in Fig. S1 is markedly different from that both Ag and Ag/Au-t systems, with two broader peaks at 390 nm and 422 nm. The absence of such absorption features in the spectrum of the Ag/Au-t clusters (Fig.1b) indicates that the population of Au clusters in the sample is negligible.


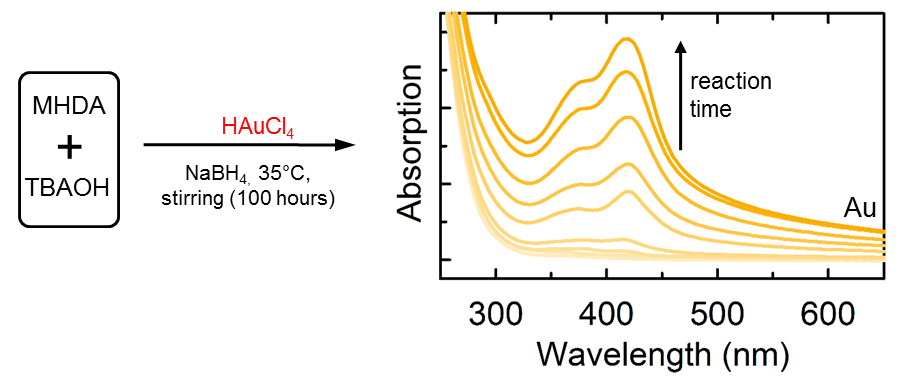


**Figure S1.** Synthesis route for the preparation of Au clusters and the correspondent absorption spectrum of aliquots of the reaction medium as function of time.

1. **ESI-MS analysis of Ag/Au-t and control Ag clusters**


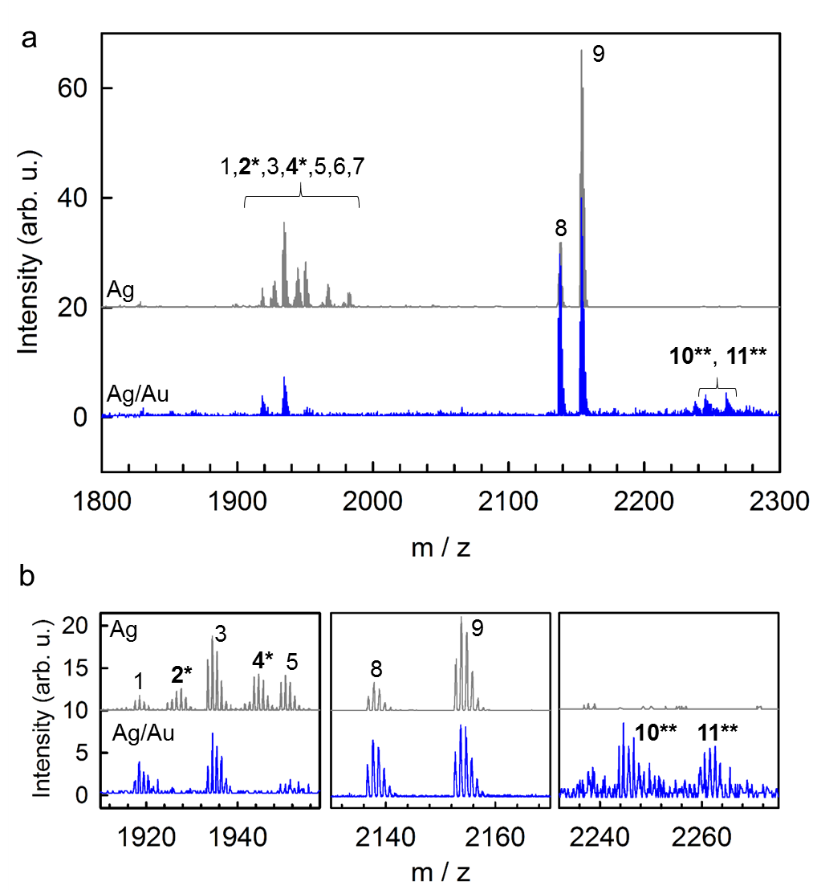


**Figure S2**. **ESI-MS spectrum of Ag and Ag/Au-t clusters.** a) Positive mode ESI**-**MS spectra of metal clusters in water. The peaks are ascribed to Ag_3_ clusters (**2***, **4***), to Ag_3_Au_3_ bimetallic clusters (**10****, **11****) and to capping ligand adducts (1, 3, 5, 6, 7, 8, 9). The details on the peaks assignment are reported in Table 1. b) Enlargement of the *m/z* ranges where the peaks corresponding to Ag clusters (left panel), Ag/Au-t clusters (right panel) and to the principal ligand adducts (middle panel) can be detected. In the case of the bimetallic system the lower signal-to-noise ratio leads to a lower resolution of the isotopic distributions.

**Table S1**. **ESI-MS analysis data of Ag and Ag/Au-t.** *m/z*-values and chemical formula of the monometallic and bimetallic clusters and the adduct species extracted from the mass spectrum reported in Fig. S3.

| **Peak** | **Chemical Species** | **Theoretical**  **average m/z** | **Experimental**  **average m/z** |
| --- | --- | --- | --- |
| **1** | [(MHDA)_4_(TBA)_3_Na_2_]^+^ | 1919.26 | 1918.62 |
| **2*** | **[Ag_3_(MHDA)_2_(TBA)_4_(NO_3_)]^+^** | 1928.41 | 1927.29 |
| **3** | [(MHDA)_4_(TBA)_3_Na_2_O]^+^ | 1935.26 | 1935.12 |
| **4*** | **[Ag_3_(MHDA)_2_(TBA)_4_(NO_3_)O]^+^** | 1944.41 | 1944.69 |
| **5** | [(MHDA)_4_(TBA)_3_Na_2_O_2_]^+^ | 1951.26 | 1950.99 |
| **6** | [(MHDA)_4_(TBA)_3_Na_2_O_3_]^+^ | 1967.26 | 1967.20 |
| **7** | [(MHDA)_4_(TBA)_3_Na_2_O_4_]^+^ | 1983.26 | 1983.07 |
| **8*** | [(MHDA)_4_(TBA)_4_Na]^+^ | 2138.74 | 2138.35 |
| **9** | [(MHDA)_4_(TBA)_4_NaO]^+^ | 2154.74 | 2154.39 |
| **10**** | **[Ag_3_Au_3_(MHDA)_2_(TBA)_3_(BH_4_)_2_]^+^** | 2244.53 | 2245.12 |
| **11**** | **[Ag_3_Au_3_(MHDA)_3_(TBA)_2_H_2_]^+^** | 2260.53 | 2261.16 |

1. **X-ray photoelectron spectroscopy (XPS) analysis of control Ag and Au clusters.**


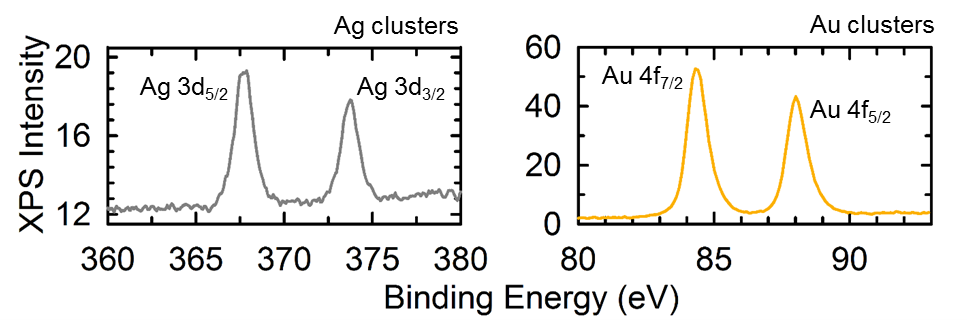


**Figure S3**. XPS spectra of Ag and Au atomic transitions measured on Ag and Au monometallic clusters.

1. **ROS scavenging activity of Ag and Ag/Au-t clusters measured by ROS-Glo™ H_2_O_2_ Assay**

The ROS scavenging activity of metal clusters has been demonstrated in solution by measuring the concentration of hydrogen peroxide (H_2_O_2_) in a saturated aqueous solution ([H_2_O_2_] = 5 mM) in the presence of the clusters over time. Triplicate samples of both metallic clusters (7 μM) were tested using ROS-Glo™ H_2_O_2_ Assay (Promega), following the manufacturers’ protocol. Briefly, an opaque white 96 wells plate was loaded with 50 μl of each sample dispersed in H_2_O_2_ (5 mM) saturated water, followed by the incubation with H_2_O_2_ substrate solution and ROS-Glo detection solution. After 20 min incubation at room temperature, the luminescence was measure by GloMax Discover (Promega) plate reader. The luminescence was recorded continuously every 15 minutes for 5 hours. After 5 hours, the H_2_O_2_ concentration is reduced to half of its initial value, while no changes are observed in the control sample without the metal cluster.

**Figure S4**. Evolution in time of the concentration of the hydrogen peroxide in a H_2_O_2_ saturated (5 mM) aqueous dispersion in presence of Ag/Au-t and Ag metal nanoclusters (7 μM). The control sample (CTR) does not contain metal nanoclusters.

1. **Optical stability in ROS solutions**

**Figure S5**. Integrated PL intensity of Ag/Au-t measured over time in water at increasing H_2_O_2_ concentrations. The excitation wavelength is 355 nm, the values are normalized for the respective intensity at t=0 s.
